# Electrochemical Behavior and Electrodeposition of Nanostructured Rhodium from Low-Temperature Carbamide and Acetamide Containing Melts

**DOI:** 10.1186/s11671-017-2108-7

**Published:** 2017-05-11

**Authors:** Svetlana Kochetova, Anastasiya Savchuk, Dmytro Shakhnin, Viktor Malyshev

**Affiliations:** Vernadsky Institute of General and Inorganic Chemistry, 32/34 Palladin Ave., Kyiv, 03142 Ukraine

**Keywords:** Nanoparticles, Nanocoatings, Rhodium, Electrochemical behavior, Synthesis, Low-temperature melts

## Abstract

The electrochemical behavior of rhodium at low-temperature carbamide-chloride and acetamide-chloride melts was investigated. It was found that, during rhodium anodic dissolution in carbamide and acetamide containing chloride melts, mixed complexes [Rh(NH_3_)_4_Cl_2_]^+^ of quasi-octahedral symmetry D_4h_ are produced. The composition and structure of nascent complex ions have been studied. During electrochemical reduction of [Rh(NH_3_)_4_Cl_2_]^+^ complexes, the synthesis of Rh nanoparticles, as well as Fe, Cu, and Mo nanocoatings, were realized.

## Background

Nanoparticles and materials based on them exhibit unique electrical, chemical, magnetic, optical, catalytic, and other properties. That is why such studies receive considerable attention [Bychkova [1], Gusev and Rempel [2], and Beloglazkina et al. [3]]. In connection with a wide area of nanocomposite applications in modern technologies and material science, methods are developing for their synthesis [Olenin and Lisichkin [4]]. Of great importance in the solution of this problem are the methods of electrochemical reduction of metal complexes in low-temperature melts. Development of methods of synthesis of metal nanoparticles in low-temperature ion-organic melts with the use of complex compounds contributes to the creation of new technologies of their production.

This paper is devoted to the electrochemical synthesis of complex rhodium containing compounds in ion-organic melts based on urea and acetamide, thorough investigation of their properties and structure using spectroscopic methods, and realization of the cathodic deposition of rhodium nanostructured coatings onto different metal substrates.

## Methods

To determine the electrochemical properties of the solvent melts and to study the electrochemical behavior of Rh in carbamide and acetamide containing melts, method of cyclic voltammetry was chosen using PI-50-1.1 potentiostat. As the anode, the investigated metal plate was used, and the auxiliary electrode was a platinum rod. As the reference electrode, Ag/Ag + half-cell was used. Studies were carried out in the temperature range 80–130 °C under argon.

To determine the state of metal ions in the melts after the electrochemical dissolution and also to determine the structure and composition of the complexes formed with the components of the melt, the following spectroscopic methods were used: electronic absorption spectroscopy (EAS) during electrolysis (with Specord UV/VIS), infrared spectroscopy of rapidly solidified after electrolysis melts (with Specord M-80), and gas chromatography (with LCM-80).

The obtained metallic deposit composition was determined by X-ray phase analysis method (with DRON-3 apparatus) and the deposit structure by methods of transmission electron microscopy (with JEOL-100) and of scanning electron microscopy (with REM-101).

## Results and Discussion

**Fig. 1:**
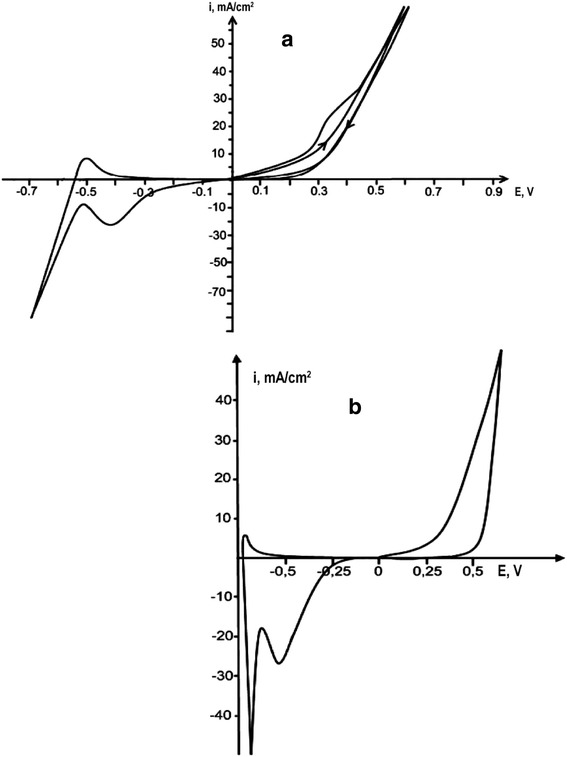
Cyclic voltammograms of rhodium electrode vs platinum rod in the melt urea-NH_4_Cl (**a**), *V*_*p*_ = 0.1 V/s, and acetamide-NH_4_Cl (**b**), *Vp* = 0.1 V/s

The anodic rhodium dissolution was carried out in the individual urea melt and in the eutectic melt urea-NH_4_Cl (16.8 mol. %) (*T* = 130 °C). In the individual molten urea, dissolution of metal is complicated by passivation, and, due to a weak electrical conductivity of the melt, increase of cathodic and anodic currents is poorly expressed. Addition of NH_4_Cl to urea increases the electrical conductivity of the melt which leads to the polarization curves clearly displaying all the electrode processes. In carbamide-chloride melt, electrochemical dissolution of rhodium proceeds initially without restrictions; however, already during the second cycle, the passivation of the anode takes place which is shown by the appearance of the shoulder at the anode part of the cyclic voltammogram (Fig. 1a). Weight losses of rhodium anode in urea-chloride melt increase as compared with the individual urea, and melt staining intensity increases and reaches a deep yellow-brown color. Rhodium electrochemical dissolution is accompanied by the formation of ions Rh (III).

Rhodium anodic dissolution was also carried out in the acetamide melt at 108 °C and in acetamide-NH_4_Cl melt at 80 °C. Polarization curves in acetamide melt are blurred, so the electrode processes cannot be correctly interpreted. Adding ammonium chloride to acetamide to form acetamide-NH_4_Cl (22.3 mol.%) melt eutectic composition [Tumanova et al. [5]] allowed us to obtain polarization curves which clearly show the electrochemical processes of the rhodium anode dissolution and of the reduction of its ions. Rhodium is dissolving in acetamide-chloride melt without passivation (Fig. 1b). Its dissolution rate (4.7∙10^−5^) is greater than in urea-chloride melt, while the specific conductivity of the urea-ammonium chloride melt (0.06 ohm^−1^cm^−1^) higher than of the acetamide-chloride one (0.015 ohm^−1^cm^−1^) [Tumanova et al. [6]]. This result can be explained by the absence of electrode passivation during the electrodissolution in acetamide-chloride melt. Upon dissolution, rhodium is transferred into the melt as Rh (III) ions.

The most stable oxidation state of rhodium is Rh (III) with the electron configuration [Kr] (4d)^6^. For Rh (III) complex compounds characterized by the formation of octahedral configuration, the main state for octahedral complexes of Rh (III) is ^1^A_1g_(t_2g_)^6^, and a singlet excited state is ^1^T_1g_ and ^1^T_2g_ relating to the configuration (t_2_g)^5^(eg).

**Fig. 2:**
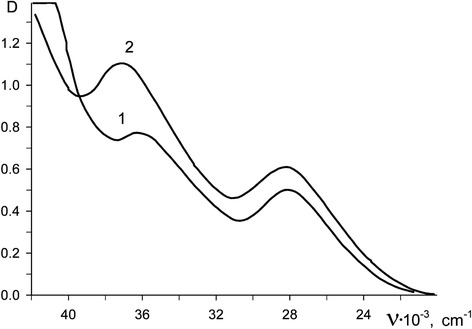
Electron spectra of the complex Rh (III) ions in chloride-acetamide (*1*) and urea-chloride (*2*) melts at 120 °C

During anodic dissolution of Rh in the urea-chloride melt, bands at 28,000 cm^−1^ and 37,000 cm^−1^ appear at electron spectra, and in acetamide-chloride melt—at 28,000 cm^−1^ and 36,000 cm^−1^ (Fig. 2). In the IR spectra of quickly cooled samples, the presence of vibrations ν(Rh-N) at 480 cm^−1^ and ν(Rh-Cl) at 330 cm^−1^ was found. In accordance with the literature data [Liver [7], Volkov and Yatsimirskiy [8], and Nakamoto [9]], this is characteristic for the formation in carbamide and acetamide containing melts of quasi-octahedral complexes [Rh(NH_3_)_4_Cl_2_]^+^ of D_4h_ symmetry.

The following spectroscopic parameters were obtained for the complexes [Rh(NH_3_)_4_Cl_2_]^+^ in urea-chloride and chloride-acetamide melts: 10 Dq = 31000 (30650) cm^−1^; *B* = 562 (500) cm^−1^; *b* = 0.78 (0.70). The strength of ligand field in the mixed complexes [Rh(NH_3_)_4_Cl_2_]^+^ is weaker than in pure ammonia complexes [Rh(NH_3_)_6_]^3+^, where 10 Dq = 33200 cm^−1^ [Galus [10]], which confirms again the formation in these melts during the rhodium anodic dissolution of mixed complexes [Rh(NH_3_)_4_Cl_2_]^+^.

**Fig. 3:**
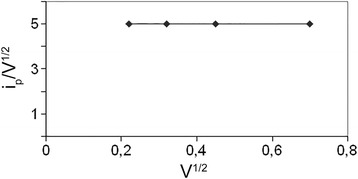
Dependence *i*_*p*_/V^1/2^ from V^1/2^ for rhodium cathode vs platinum rod in molten mixture urea-NH_4_Cl

**Fig. 4:**
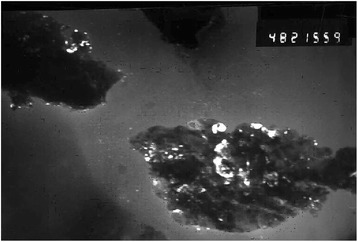
Micrograph of rhodium nanoparticles (10–22 nm) obtained at the platinum cathode in molten mixture urea-NH_4_Cl (JEOL-100)

Complex compound [Rh(NH_3_)_4_Cl_2_]^+^ in the investigated melts is electroactive, and its reduction is observed in the cathodic part of cyclograms as a maximum shown in Fig. 1a, b. Determination of kinetic parameters of the Rh (III) reduction process was carried out by a conventional method. Main criteria for the diffusion control and reversibility of the process were calculated according to the equations given in the monograph of Galus [10]. Polarization curves were recorded in a wide range of polarization rates: 0.05, 0.1, 0.2, 0.5, and 1 V/s. Dependence *i*_*p*_/V^1/2^ from V^1/2^ (Fig. 3) is rectilinear and parallel to the x-axis which shows that the process proceeds in the diffusion mode, but since there is a dependence of *i*_*p*_ on V^1/2^. The diffusion coefficient of rhodium ions in the urea-chloride and chloride-acetamide melts is determined by their respective conductivity, and its value range is within 8.4∙10^−6^ cm^2^/s for these systems. The reduction of rhodium ions occurs irreversibly in the diffusion mode in one step to the free metal. Micrograph of rhodium deposit obtained is shown in Fig. 4.

**Fig. 5:**
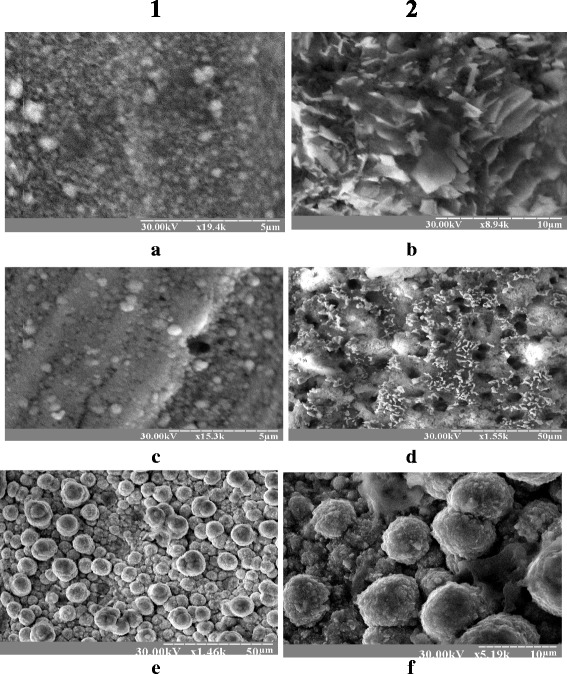
Appearance of the Rh nanocomposites at the surface of Fe (**a**, **b**), Cu (**c**, **d**), and Mo (**e**, **f**) after electrolysis in (*1*) carbamide- and (*2*) acetamide-chloride melt with the soluble Rh anode (obtained using REM-101 microscope)

The size of the Rh deposit particles is within 10–22 nm, but a significant portion of it is aggregated. The average size of the rhodium crystallites was evaluated by physical expansion of peaks and is about 5 nm. This indicates the formation of rhodium nanocomposites on the surface of metallic Fe, Cu, and Mo in the investigated melts. Rhodium coatings obtained from melts on the basis of urea and acetamide with soluble rhodium anode are uniform, of gray color, and 6.1-micron thick (Fig. 5). During Rh coating deposition, 0.05–0.7 g of metal was obtained depending on electrolysis time. Thus, the current yield was 85–90%.

## Conclusions

Thus, the electrochemical behavior of rhodium was studied in melts based on urea and acetamide. Anodic dissolution of metal in these electrolytes is accompanied by the passivation with the formation of Rh (III) complexes which are reduced in the diffusion mode, in one step to metal. The synthesis of Rh nanoparticles and nano-coatings at Fe, Cu, and Mo surface was realized.

## Abbreviations

EAS: Electronic absorption spectroscopy
